# Supplementary material for: Splice-Junction-Based Mapping of Alternative Isoforms in the Human Proteome
Source: Cell Rep. Author manuscript; Available in PMC 2020 Jan 15. (PMC6961840; doi:10.1016/j.celrep.2019.11.026)
Supplement: 2 [file NIHMS1546469-supplement-2.zip › DF1/PXD010154/Ovary/Ovary_7_ARID1B_SQPPLAPGKPNHEDLNLIQQERPSSLPVEVLASEDAAFGLK.pdf]

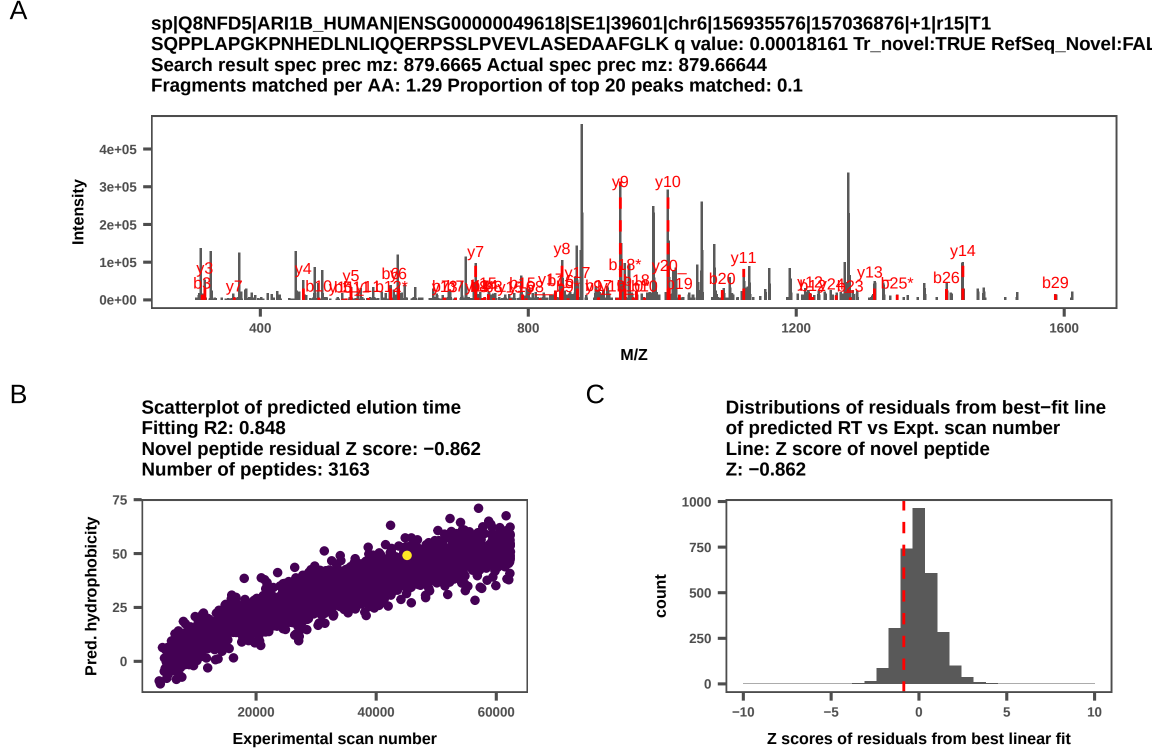
A
sp|Q8NFD5|ARI1B_HUMAN|ENSG00000049618|SE1|39601|chr6|156935576|157036876|+1|r15|T1
SQPPLAPGKPNHEDLNLIQQERPSSLPVEVLASEDAAFGLK q value: 0.00018161 Tr_novel:TRUE RefSeq_Novel:FAL
Search result spec prec mz: 879.6665 Actual spec prec mz: 879.66644
Fragments matched per AA: 1.29 Proportion of top 20 peaks matched: 0.1
Intensity
4e+05
3e+05
2e+05
1e+05
0e+00
y9
y10
y7
y8
y3
b3
y7
y4
y5
b18*
y20
y11
b20
b19
y14
y13
b26
b25*
b29
400
800
1200
1600
M/Z
B
Scatterplot of predicted elution time
Fitting R2: 0.848
Novel peptide residual Z score: −0.862
Number of peptides: 3163
Pred. hydrophobicity
75
50
25
0
20000
40000
60000
Experimental scan number
C
Distributions of residuals from best−fit line
of predicted RT vs Expt. scan number
Line: Z score of novel peptide
Z: −0.862
count
1000
750
500
250
0
−10
−5
0
5
10
Z scores of residuals from best linear fit
